# Creating and validating an instrument to identify the workload at an oncology and hematology outpatient service

**DOI:** 10.1590/S1679-45082014AO2996

**Published:** 2014

**Authors:** Lelia Gonçalves Rocha Martin, Raquel Rapone Gaidzinski

**Affiliations:** 1 Hospital Israelita Albert Einstein, São Paulo, SP, Brazil.; 2 Escola de Enfermagem, Universidade de São Paulo, São Paulo, SP, Brazil.

**Keywords:** Workload, Oncology Service, Hospital, Oncologic nursing, Personnel Downsizing

## Abstract

**Objective:**

Construct and to validate an instrument for measuring the time spent by nursing staff in the interventions/activities in Outpatient Oncology and Hematology, interventions based on Nursing Interventions Classification (NIC), for key areas of Pediatric Oncology and Oncology Nursing.

**Methods:**

Cross-sectional study divided into two steps: (1) construction of an instrument to measure the interventions/Nursing activities and (2) validation of this instrument.

**Results:**

We selected 32 essential interventions from NIC for Pediatric Oncology and Oncology Nursing areas. The judges agreed with removing 13 and including 6 interventions in the instrument, beyond personal activity.

**Conclusion:**

The choice of essential interventions from NIC is justified by the gain time on research.

## INTRODUCTION

Identifying the workload is the key for determining the number of Nursing staff, according to the Canadian Nurses Association (CNA).^(1)^ At outpatient services, emergency rooms and primary care units, for instance, the workload can be calculated as the product of time spent in interventions and Nursing activities and the number of patients who received those interventions/care.^(2)^ At an outpatient unit, nursing staff workload is influenced by several factors, such as: patient characteristics (patient classification systems), characteristics of the role of nursing staff (competencies, skills and attitudes) and number of patients who require care. Due to frequent changes in schedules and patient flow, nursing staff workload at outpatient services is often less predictable.^(3)^

In order to determine the workload at inpatients units we need to classify patients according to their level of dependence on nursing staff. A Patient Classification System should be adopted ^(2, 4)-6)^.

Over several decades nursing managers have developed a wide range of tools to measure workloads at inpatients units. However such tools are not easily adaptable to outpatient units. This shortage has been leading institutions to use some metrics, such as the number of physicians, clinical profile, number of scheduled patients and number of procedures to determine the appropriate number of staff. Nevertheless, those metrics do not reflect the number of nursing staff that is required to support nursing care practice.^(7)^

Brazilian researchers^(8)-16)^ have been using the taxonomy proposed by the Center for Nursing Classification and Clinical Effectiveness (CNC), entitled Nursing Interventions Classification (NIC) as a reference to identify nursing care interventions, with the purpose of determining nursing workload.^(17)^

The NIC organized essential interventions for 45 specialties, that is, those that refer to a limited set of core interventions and that define the nature of a specialty. Those are the most frequent, predominant or essential interventions for the role of a specialist nurse.^(17)^

Determining the appropriate number of nursing staff according to the patients’ needs in outpatient care is vital in order to provide safe care, for an optimal benefit-cost ratio and for the quality of the environment. Continuous assessment of the work environment and the implementation of consistent, reliable and valid measures is critical in order to anticipate and justify personnel needs.^(18)^

The workload is always the most important indicator when determining the number of nursing staff. It is regarded as a powerful management tool because it demonstrates how important it is to balance the number of staff both in quantitative and qualitative terms when providing care to health service users. The workload as an indicator supports administrative and policy decisions made by nurses. It also contributes effectively to the negotiations surrounding the number of nursing staff conducted with healthcare organization managers.^(18, 19)^ A previously validated instrument is required to identify this workload.

Our study began with the following question: which activities interfere with the workload of a nursing team in an outpatient unit? Based on this question, we developed the following hypothesis: essential nursing interventions in the field of Oncology listed in the NIC could provide a reference for creating an instrument to determine the workload within an Oncology and Hematology outpatient environment.

## OBJECTIVE

To create and validate an instrument to measure time spent by the nursing team on interventions/activities at an Oncology and Hematology Outpatient Service, based on the interventions listed in the Nursing Interventions Classification (NIC) for the essential areas of Pediatric Oncology and Oncology Nursing.

## METHODS

This cross-sectional study was conducted at the Escola de Enfermagem da Universidade de São Paulo, upon approval by the Research Ethics Committee under number 170,263/12. Research data were organized and processed in two stages.

### First stage: creating the instrument

First we selected the interventions proposed by the NIC for essential Nursing areas, namely Pediatric Oncology and Oncology Nursing, which are considered representative of outpatient nursing practice in Oncology and Hematology.

The interventions we selected were listed in ascending order, according to domains, with their respective NIC code. This distribution aimed at optimizing the identification of interventions during the following stage, resulting in a workshop.

We created an instrument consisting of 32 interventions distributed across 6 domains out of 7 listed in the NIC: Physiological: Complex, Behavioral, Physiological: Basic, Health System, Safety and Family. There were no interventions within the Community domain.

In the instrument, researchers asked respondents to assess each activity in terms of the representativeness of care assistance and management practices followed by Nursing professionals at an Oncology and Hematology Outpatient Service. Furthermore, we tested the relevance of interventions, according to the NIC’s definition. We also investigated whether other interventions should be included or excluded. At the end of the instrument, we provided eight lines so that respondents could include any other intervention or activity.

Research details were shown to judges using PowerPoint slides ten minutes before the instrument’s assessment. At that point, we explained the goals of the workshop: to analyze and validate nursing interventions selected according to the NIC, in addition to answering any further questions.

There were two stages during the workshop. First participants were given a chance to reread the instrument and give their opinion on it. The second stage consisted of a consensus on answers.

Five days before the workshop, the instrument was sent (Chart 1) to the judges by email, together with a letter of instructions.

### Second stage: validating the instrument

Validation assessment means identifying to what extent the instrument is appropriate to measure what it intends to measure.^(20)^ In this research we used content validation, which involves judgment from experts with extensive professional experience regarding the elements contained in the instrument and who are critical of the representativeness of that which we intend to measure.^(21, 22)^

We employed the Delphi method to analyze data. This consists of requesting, collecting, arranging and analyzing data regarding a particular phenomenon. Such data result from the opinions of experts in the proposed field of study. This method involves an interactive questionnaire that is submitted several times to the group. They answer the questionnaire, and the aim is to search for similar opinions on the result.^(23, 24)^

The first stage in the Delphi method was selection of judges. We used the following criteria: judges had to be nurses or nursing technicians with at least five years’ experience at an Oncology Outpatient Service and/or with at least 5 years’ experience using the NIC; they also had to agree to take part in the workshop. All of them agreed to take part in this stage.

We proposed the best dates, times and locations for the group of judges. After the judges were chosen, we sent them information about the workshop meeting, which was expected to last 4 hours.

Interventions were deemed validated when judges reached an agreement rate ≥70%.^(22)^

Data were stored in a database created for this research using Microsoft Excel 2013.

Validated interventions were grouped into a single instrument containing interventions that contributed to the workload of the Nursing team in an Oncology and Hematology Outpatient Service.

## RESULTS

We selected essential NIC interventions for the following specialties: Pediatric Oncology and Oncology Nursing. This selection resulted in 32 interventions, with their respective codes and definitions, as shown in chart 2.

**Figure 1:**
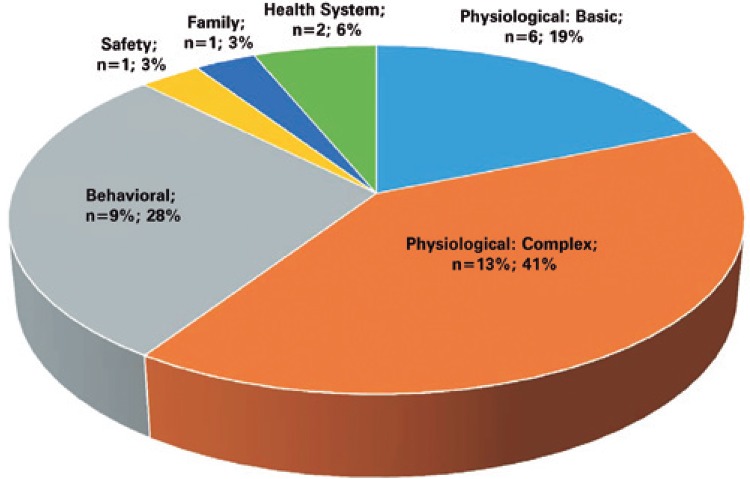
Distribution of the Nursing Interventions Classification domains (NIC), from interventions selected for the Oncology and Hematology Outpatient Service. São Paulo, 2013

Interventions are distributed across six out of seven NIC domains (Figure 1).

The Workshop lasted four hours and was held at the Escola de Enfermagem da USP. During the workshop, each intervention was assessed in terms of the representativeness of interventions made at the Oncology and Hematology Outpatient Service, and of the relevance of interventions, according to the NIC’s definition. Furthermore, we investigated whether there were any interventions that should be included or excluded.

The workshop was led by the researcher. Six judges participated and are characterized in table 1.

**Chart 1:** Essential interventions in Pediatric Oncology and Oncology Nursing

Domain 1. Physiological: Basic. Care that supports physical functioning
Class B urinary output management: interventions to establish and maintain regular bowel and urinary voiding patterns and to manage complications due to altered patterns
Intervention: 0590 Urinary output control
Definition: maintaining an excellent urinary output pattern
Is this a representative intervention in terms of the Nursing work conducted at an Oncology and Hematology Outpatient Service?__1 ⎕ No__ 2 ⎕ Yes
Is mapping this NIC intervention relevant?__1 ⎕ No__ 2 ⎕ Yes
Would you remove this intervention?__1 ⎕ No__ 2 ⎕ Yes
Would you include any other intervention?__⎕ No__ ⎕ Yes, Which one?
Intervention: ________________________________________________

NIC: Nursing Interventions Classification.

With respect to all 32 Nursing interventions we selected, 100% of the judges agreed to exclude 13 of them and to include another 6. They also agreed on grouping and maintaining the other interventions, as well as including personal activity. This resulted in 25 interventions and 1 personal activity.

From all 25 validated interventions, 84% were part of essential interventions for specialties areas in Pediatric Oncology and Oncology Nursing.

**Figure 2:**
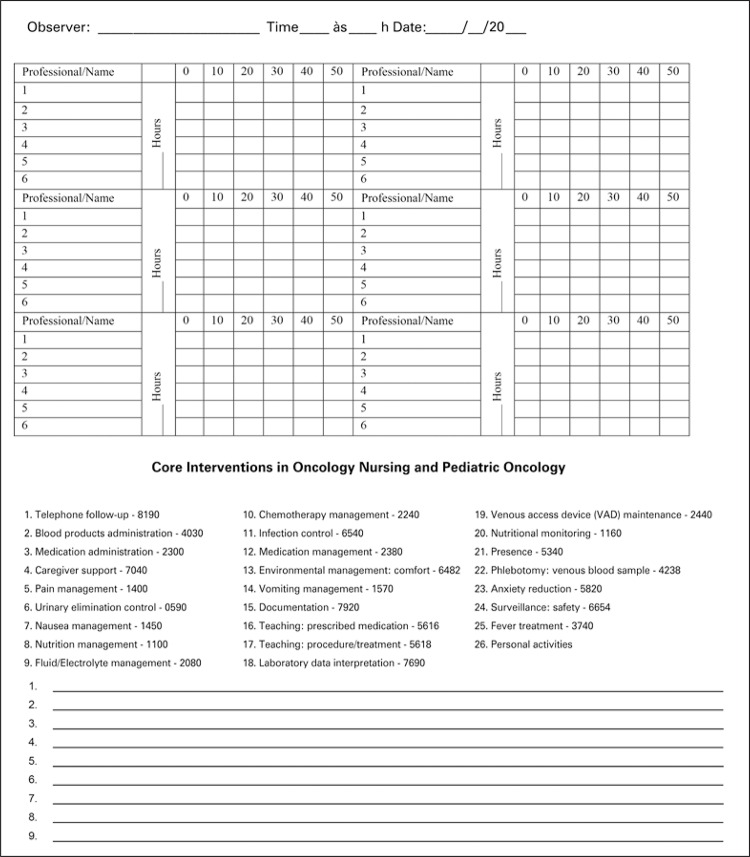
Data collection instrument

Based on the results of validated interventions and activities, we developed an instrument to track the time spent by the Nursing team of an Oncology and Hematology Outpatient Service performing their nursing activities/interventions. In order to optimize data collection, interventions were listed alphabetically, with their respective NIC codes, and numbered in ascending order. Work days were divided into columns of 10-minute intervals in order to record the intervention/activity performed (Figure 2).

## DISCUSSION

Our selection based on essential NIC interventions for Nursing in Pediatric Oncology and Oncology Nursing, which we used as a starting point for creating the instrument, resulted in 84% of interventions being validated at the workshop. This confirms the hypothesis that essential interventions for specialties provide reference for research. In addition, they facilitate the identification of interventions that have an impact on Nursing teams’ workload and on reducing time spent by researchers when selecting interventions.

The workshop method was also used in two other instances:^(21)^ to validate nurse activities at a chemotherapy center and to map those activities in Nursing interventions, according to the NIC.

The use of NIC interventions as a reference in Oncology Outpatient Service research papers has been adopted by several authors^(25, 26)^ and in studies conducted in other areas in order to measure the workload of Nursing teams.^(27)^

In an outpatient setting, time is crucial when providing safe care. The study of interventions is beneficial because it reflects all activities. Therefore, the choice for essential interventions for Nursing specialties in Pediatric Oncology and Oncology Nursing, under the Nursing Interventions Classification (NIC), and not for activities, is justified by the time saved on research.

De Souza et al.^(26)^ initiated their research based on the activities conducted by nurses at a Chemotherapy Outpatient Service in a hospital specializing in Oncology. They used semi-structured interviews, document analysis and a questionnaire. After that they mapped activities into interventions, according to the NIC.

Interventions mapped in this study resulted in 32 interventions distributed across 6 domains: Physiological: Complex, Physiological: Basic, Behavioral, Health System, Family and Safety. They did not use personal activities because they believed such activities could hinder comparisons with other studies, since activities may vary between different institutions. However, during the workshop judges suggested including personal activities, without specifying what type.

Other researchers^(26)^ have achieved different results. At a chemotherapy center, 35 interventions and 48 activities were identified. Personal activities were among them, described as meal or restroom breaks. Interventions were organized into five domains and unlike our study they did not include the family domain. Other researchers^(28)^ from a diagnostic imaging center identified 32 interventions and 92 activities.

During the workshop, participants realized the importance of balancing knowledge from the NIC and practical experience. This was also noted by other authors,^(28)^ who identified the advantages of this method, since it provides a critical analysis for improving the proposed instrument.

The 25 interventions and 1 activity from the workshop validation created an instrument to measure workload. Nursing interventions are listed alphabetically with their respective NIC codes. Work days were divided into columns in 10-minute intervals. We included fields for up to six observers. This same formatting has been used^(28)^ to propose an instrument to measure the working hours of Nursing professionals at a diagnostic imaging center. However, there were no fields in the instrument for six simultaneous observers, only space reserved for two of them.

## CONCLUSION

It is clear that using essential interventions for Nursing specialties in Pediatric Oncology and Oncology Nursing as a reference optimized the time spent on research. However, other specialties should also be assessed and possibly included in the instrument, such as Outpatient Care Nursing, Palliative Care Nursing, Occupational Health Nursing and Emergency Nursing.

The instrument we developed was validated in a workshop, during which the instrument was considered appropriate to measure the workload of the Nursing team in an Oncology and Hematology Outpatient Service. However, it has some limitations. It needs to be applied in nursing care practice so that its reliability can be assessed and so that its degree of precision can be established. In order words, we still need to find out to what extent the proposed instrument reflects data identified in it.

**Chart 2:** List of 32 Nursing interventions selected according to the Nursing Interventions Classification (NIC) for an Oncology and Hematology Outpatient Service

Code	Mapped Interventions	Definitions
8190	Telephone follow-up	Providing results of tests, evaluating patient’s response and determining potential for problems as a result of previous treatment, examination or testing, over the telephone
2210	Analgesic administration	Use of pharmacologic agents to reduce or eliminate pain
4030	Blood products administration	Administration of blood or blood products and monitoring of patient’s reactions
2313	Medication administration: intramuscular	Preparing and giving medications via the intramuscular route
2304	Medication administration: oral	Preparing and giving medications by mouth
2314	Medication administration: intravenous (IV)	Preparing and giving medications via the intravenous route
7040	Caregiver support	Provision of the necessary information, advocacy and support to facilitate primary patient care by someone other than a healthcare professional
5420	Spiritual support	Assisting the patient to feel balance and connection with a greater power
4430	Therapeutic play	Purposeful and directive use of toys or other materials to assist children in communicating their perception and knowledge of their world and to help in gaining mastery of their environment.
1400	Pain management	Alleviation of pain or a reduction in pain to a level of comfort that is acceptable to the patient
0590	Urinary output control	Maintaining an excellent urinary output pattern
1450	Nausea management	Prevention and alleviation of nausea
1100	Nutrition management	Providing and promoting a balanced intake of nutrients and fluids
2240	Chemotherapy management	Assisting the patient and family members to understand the action and minimize side effects of antineoplastic agents
6540	Infection control	Minimizing the acquisition and transmission of infectious agents
2380	Medication management	Facilitating safe and effective use of prescription and over-the-counter drugs
6482	Environmental management: comfort	Manipulation of the patient’s surroundings for promotion of optimal comfort
1570	Vomiting management	Prevention and alleviation of vomiting
4120	Fluid management	Promotion of fluid balance and prevention of complications resulting from abnormal or undesired fluid levels
2080	Fluid/Electrolyte management	Regulation and prevention of complications from altered fluid and/or electrolyte levels
5230	Coping enhancement	Assisting a patient to adapt to perceived stressors, changes, or threats which interfere with meeting life demands and roles.
5566	Parent education: childrearing family	Assisting parents to understand and promote the physical, psychological, and social growth and development of their toddler, preschool, or school-aged child/children
4235	Phlebotomy: cannulated vessel	Aspirating a blood sample through an indwelling vascular catheter for laboratory tests
5820	Anxiety reduction	Minimizing apprehension, dread, foreboding, or uneasiness related to an unidentified source of anticipated danger
5880	Calming technique	Reducing anxiety in patient experiencing acute distress
4200	Intravenous therapy (IV)	Administration and monitoring of intravenous fluids and medications
3740	Fever treatment	Management of a patient with hyperpyrexia caused by non-environmental factors

**Table 1:** Characteristics of judges who took part in the workshop to validate interventions chosen according to the Nursing Interventions Classification (NIC) on February 4, 2013. São Paulo, 2013

Characteristics	n (%)
Sex	
Female	5 (83.3)
Male	1 (16.7)
Age	
≥30 years	6 (100.0)
Occupation	
Professor	3 (50.0)
Nursing Manager	1 (16.7)
Senior Nurse	1 (16.7)
Nursing Technician	1 (16.7)
Highest Degree	
PhD	2 (33.3)
Master	2 (33.3)
Specialist	1 (16.7)
Nursing Degree	1 (16.7)
Years of experience since graduation (average)	
<1	1 (16.7)
≥ 5-10	1 (16.7)
≥10	4 (66.7)
Average total years of experience in Oncology	
0	2 (33.3)
7	1 (16.7)
10	1 (16.7)
12	1 (16.7)
15	1 (16.7)
Average total years of experience in NIC	
0	1 (16.7)
<1	2 (33.3)
6	1 (16.7)
7	2 (33.3)
