# Supplementary material for: Creating and validating an instrument to identify the workload at an oncology and hematology outpatient service
Source: Einstein (Sao Paulo). 2014 Jul-Sep;12(3):323–9. doi: 10.1590/S1679-45082014AO2996 (PMC4872944; doi:10.1590/S1679-45082014AO2996)
Supplement: Translation

# Translation: Construção e validação de instrumento para identificação de carga de trabalho em ambulatório de oncologia e hematologia

**Authors:** Lelia Gonçalves Rocha Martin, Raquel Rapone Gaidzinski

**Affiliations:** 1 Hospital Israelita Albert Einstein, São Paulo, SP, Brasil.; 2 Escola de Enfermagem, Universidade de São Paulo, São Paulo, SP, Brasil.

**Keywords:** Carga de trabalho, Serviço hospitalar de Oncologia, Enfermagem oncológica, Downsizing organizacional

## Abstract

**Objetivo:**

Construir e validar um instrumento para medida de tempo despendido pela equipe de enfermagem nas intervenções/atividades do Ambulatório de Oncologia e Hematologia, com base nas intervenções da Classificação das Intervenções de Enfermagem (NIC - *Nursing Interventions Classification*), para áreas essenciais de Oncologia Pediátrica e Enfermagem Oncológica.

**Métodos:**

Estudo transversal, dividido em duas etapas: (1) construção do instrumento para medida das intervenções/atividades de Enfermagem e (2) validação desse instrumento.

**Resultados:**

Foram selecionadas 32 intervenções essenciais da NIC para as áreas de Oncologia Pediátrica e Enfermagem Oncológica. Os juízes concordaram com a remoção de 13 intervenções e a inserção de 6, além da atividade pessoal.

**Conclusão:**

A escolha por intervenções essenciais para as áreas de especialidades da NIC é justificada pelo ganho de tempo em pesquisa.

## INTRODUÇÃO

A identificação da carga de trabalho é a chave para a determinação do quadro de profissionais de Enfermagem, segundo a *Canadian Nurses Association* (CNA).^(1)^ Em ambulatórios, prontos-socorros e Unidades Básica de Saúde, por exemplo, o cálculo da carga de trabalho pode ser obtido pelo produto do tempo médio despendido na realização das intervenções e nas atividades de Enfermagem pela quantidade de pacientes assistidos naquela intervenção/atividade.^(2)^ Em uma unidade ambulatorial, a carga de trabalho de Enfermagem é influenciada por diversos fatores, como: características do paciente (sistemas de classificação de pacientes), características do papel da Enfermagem (competências, habilidades e atitudes) e número de pacientes que requerem cuidados. Devido às frequentes alterações de programação e de fluxo de pacientes, a carga de trabalho da Enfermagem ambulatorial é, muitas vezes, menos previsível.^(3)^

Para conhecer a carga de trabalho em unidades de internação, é necessário classificar os pacientes de acordo com o grau de dependência da equipe de Enfermagem, devendo-se adotar um Sistema de Classificação do Paciente ^(2, 4)-6)^

Por várias décadas, gestores de Enfermagem desenvolveram uma grande variedade de ferramentas de medição de carga de trabalho em unidades de internação. Entretanto, tais ferramentas não são facilmente adaptáveis às unidades ambulatoriais. Essa escassez tem levado as instituições a utilizarem métricas, como número de médicos, quadro clínico, número de pacientes agendados e volumes de procedimentos, para determinar o número adequado de pessoal. Porém, essas métricas não refletem o número de pessoal de Enfermagem necessário para apoiar a prática assistencial.^(7)^

Para identificar as intervenções de cuidado desenvolvidas pela Enfermagem, com o objetivo de conhecer a carga de trabalho, pesquisadores brasileiros^(8)-16)^ têm utilizado como referencial a taxonomia proposta pelo *Center for Nursing Classification and Clinical Effectiveness* (CNC), denominada Classificação das Intervenções de Enfermagem (NIC - *Nursing Interventions Classification*).^(17)^

A NIC organizou as intervenções essenciais para 45 áreas de especialidades, ou seja, aquelas que se referem a um conjunto limitado de intervenções centrais e que definem a natureza de uma especialidade. São estas as intervenções de maior frequência, predominantes, ou as essenciais para o papel do enfermeiro especialista.^(17)^

Dimensionar a equipe de Enfermagem de forma adequada às necessidades dos pacientes assistidos em ambulatórios é fundamental para a prestação de cuidados seguros, para um bom custo-benefício e para a qualidade do ambiente. A avaliação contínua do ambiente de trabalho e o estabelecimento de medidas consistentes, confiáveis e válidas são fundamentais para prever e justificar as necessidades de pessoal.^(18)^

A carga de trabalho é sempre o indicador mais importante na realização do dimensionamento de pessoal. Ela é considerada um poderoso instrumento de gestão, por ser capaz de demonstrar a importância da adequação quantitativa e qualitativa do quadro de pessoal na prestação de cuidados aos usuários dos serviços de saúde. A carga de trabalho, como indicador, subsidia as decisões administrativas e as políticas dos enfermeiros, além de contribuir efetivamente para a negociação do quadro de profissionais com os administradores das organizações de saúde.^(18, 19)^ Para identificá-la, é necessário um instrumento que tenha sido anteriormente validado.

Este estudo partiu do questionamento: quais as atividades que interferem na carga de trabalho da equipe de Enfermagem de uma unidade ambulatorial? Com base nesse questionamento, foi formulada a seguinte hipótese: as intervenções de Enfermagem essenciais para a área de Oncologia apresentadas na NIC podem ser o referencial para construção de um instrumento que possa identificar a carga de trabalho em um ambiente ambulatorial de Oncologia e Hematologia.

## OBJETIVO

Construir e validar um instrumento para medida de tempo despendido pela equipe de enfermagem nas intervenções/atividades do Ambulatório de Oncologia e Hematologia, com base nas intervenções da Classificação das Intervenções de Enfermagem (NIC - *Nursing Interventions Classification*), para áreas essenciais de Oncologia Pediátrica e Enfermagem Oncológica.

## MÉTODOS

Trata-se de um estudo transversal ocorrido nas dependências da Escola de Enfermagem da Universidade de São Paulo, após a aprovação pelo Comitê de Ética em Pesquisa, sob número 170.263/12. Os dados da pesquisa foram organizados e processados em duas etapas.

### Primeira etapa: construção do instrumento

Foram selecionadas as intervenções propostas pela NIC para áreas essenciais de Enfermagem, a saber Oncologia Pediátrica e Enfermagem Oncológica, consideradas representativas da prática de Enfermagem ambulatorial de Oncologia e Hematologia.

As intervenções selecionadas foram listadas em ordem crescente, por domínios, com a identificação do código NIC. Essa distribuição teve o objetivo de otimizar a identificação das intervenções durante próxima etapa, que consistiu na realização de uma oficina.

Foi criado um instrumento constituído por 32 intervenções, distribuídas em 6 domínios dos 7 apresentados pela NIC: Fisiológico complexo, Comportamental, Fisiológico básico, Sistema de saúde, Segurança e Família. Não houve intervenção referente ao domínio Comunidade.

No instrumento, a pesquisadora solicitou a avaliação de cada atividade quanto à representatividade das práticas de cuidado assistenciais e gerenciais desenvolvidas pelos profissionais de Enfermagem no Ambulatório de Oncologia e Hematologia. Além disso, testou-se a pertinência da intervenção, segundo a definição da NIC. Verificou-se também a necessidade de inclusão ou exclusão de outras intervenções. Ao final do instrumento, foram disponibilizadas oito linhas para possível inclusão de qualquer intervenção ou atividade.

O detalhamento da pesquisa foi apresentado aos juízes em *PowerPoint*, 10 minutos antes da avaliação do instrumento. Nesse momento, houve um alinhamento no objetivo da oficina que foi analisar e validar as intervenções de enfermagem selecionadas segundo a *NIC*, além de esclarecimentos de dúvidas.

Durante a oficina, ocorreram duas etapas. A primeira foi a releitura e a manifestação dos participantes quanto à sua opinião do instrumento. A segunda etapa foi constituída pelo consenso das respostas.

Cinco dias antes da oficina, o instrumento foi enviado (Quadro 1) por *e-mail* aos juízes, junto de carta de orientação.

### Segunda etapa: validação do instrumento

A avaliação da validade é a identificação do grau em que o instrumento se mostra apropriado para mensurar o que supostamente deve medir.^(20)^ Nesta pesquisa, foi utilizada a validade de conteúdo, na qual há o envolvimento do julgamento por *expertises* com vasta experiência profissional em relação aos elementos contidos no instrumento e críticos quanto representatividade do que se pretende medir.^(21, 22)^

Para análise dos dados foi aplicada a técnica Delphi, que consiste em solicitar, coletar, tabular e analisar os dados referente a um fenômeno, resultante da opinião de especialistas na área do estudo proposto. Tal técnica prevê a aplicação de questionário interativo, que é apresentado por diversas vezes ao grupo, que o responde, em busca de opiniões similares sobre o resultado.^(23, 24)^

A primeira etapa da técnica Delphi foi a seleção dos juízes. Os critérios utilizados foram: ser enfermeiro ou técnico de Enfermagem com experiência mínima de 5 anos em Ambulatório de Oncologia e/ou com experiência mínima de 5 anos na utilização da NIC, e consentir em participar da oficina. Todos consentiram em participar dessa etapa.

Datas, horários previstos e local que melhor atendesse o agrupamento dos juízes foram propostos. Após a seleção, foram enviadas as informações para o encontro na oficina, com previsão de duração de 4 horas.

As intervenções foram consideradas validadas quando os juízes apresentaram um índice de concordância ≥70%.^(22)^

Os dados foram armazenados em um banco de dados elaborado para a pesquisa em *Microsoft Excel 2013*.

As intervenções validadas foram agrupadas em um instrumento contendo as intervenções que contribuíam para a carga de trabalho da equipe de Enfermagem do Ambulatório de Oncologia e Hematologia.

## RESULTADOS

Foram selecionadas da NIC as intervenções essenciais para as áreas de especialidades: Oncologia Pediátrica e Enfermagem Oncológica. Essa seleção resultou em 32 intervenções, com seus respectivos códigos e definições, conforme demonstrado no quadro 2.

As intervenções estão distribuídas em seis dos sete domínios apresentados pela NIC (Figura 1).

**Figura 1:** Distribuição dos domínios *Nursing Interventions Classification* (NIC), resultante das intervenções selecionadas para os Ambulatório de Oncologia e Hematologia. São Paulo, 2013

A Oficina ocorreu em quatro horas, nas dependências da Escola de Enfermagem da USP. Durante a oficina, cada intervenção foi avaliada em relação à representatividade das intervenções desenvolvidas em Ambulatório de Oncologia e Hematologia, e à pertinência da intervenção, segundo a definição da NIC. Além disso, foi verificada a necessidade de incluir ou excluir intervenções.

A oficina foi coordenada pela pesquisadora, composta por seis juízes, caracterizados na tabela 1.

**Quadro 1:** Intervenções essenciais para Oncologia Pediátrica e Enfermagem Oncológica

Domínio 1. Fisiológico básico: cuidados que dão suporte ao funcionamento físico
Classe B controle da eliminação urinária: intervenções para estabelecer e manter padrões regulares de eliminação intestinal e urinária e controlar complicações resultantes de padrões alterados
Intervenção: 0590 Controle da eliminação urinária
Definição: manutenção de um padrão excelente de eliminação urinária
A intervenção é representativa do trabalho de Enfermagem realizado no Ambulatório de Oncologia e Hematologia?__1 ⎕ Não__ 2 ⎕ Sim
O mapeamento dessa intervenção da NIC é pertinente?__1 ⎕ Não__ 2 ⎕ Sim
Você excluiria essa intervenção?__1 ⎕ Não__ 2 ⎕ Sim
Você incluiria alguma outra intervenção?__⎕ Não__⎕ Sim, Qual?
Intervenção: ___________________________________________________________________

NIC: Classificação das Intervenções de Enfermagem.

Das 32 intervenções de Enfermagem selecionadas, houve concordância de 100% dos juízes sobre excluir 13, inserir 6, e agrupar e manter as demais intervenções, além de incluir a atividade pessoal, resultando em 25 intervenções e 1 atividade pessoal.

Entre as 25 intervenções validadas, 84% faziam parte das intervenções essenciais para as áreas de especialidades em Oncologia Pediátrica e Enfermagem Oncológica.

Com o resultado das intervenções e atividades validadas, foi desenvolvido um instrumento para coleta dos tempos despendidos pela equipe de Enfermagem do Ambulatório de Oncologia e Hematologia, no desempenho das atividades/intervenções de Enfermagem. Para otimizar a coleta, as intervenções foram listadas em ordem alfabética, com identificação do código NIC, e numeradas em ordem crescente. A jornada de trabalho foi dividida em colunas, em intervalos de 10 minutos, para o registro da intervenção/atividade realizada (Figura 2).

**Figura 2:** Instrumento de coleta

## DISCUSSÃO

A seleção a partir das intervenções essenciais para Enfermagem em Oncologia Pediátrica e Enfermagem Oncológica da NIC, como ponto de partida na construção do instrumento, resultou em 84% das intervenções validadas na oficina, o que confirmou a hipótese de que as intervenções essenciais para especialidade constituem um referencial para pesquisa, além de facilitarem a identificação das intervenções que impactam na carga de trabalho da equipe de Enfermagem e na redução do tempo do pesquisador na seleção das intervenções.

A técnica de oficina também foi utilizada em dois momentos:^(21)^ para validar as atividades dos enfermeiros em um centro de quimioterapia e para seu mapeamento em intervenções de Enfermagem, segundo a NIC.

O uso das intervenções da NIC como referencial em trabalhos de Ambulatório de Oncologia foi adotado por vários autores^(25, 26)^ e em estudos realizados em outras áreas, com o objetivo de mensurar a carga de trabalho da Enfermagem.^(27)^

Em ambulatório, tempo é fator crucial para o desempenho da assistência prestada com garantia de segurança. O estudo das intervenções resulta em ganho, por retratar todas as atividades. Dessa forma, a escolha por intervenções essenciais, para as áreas de especialidades de Enfermagem em Oncologia Pediátrica e Enfermagem Oncológica, da Classificação das Intervenções de Enfermagem (NIC), e não de atividades, é justificada para ganho de tempo em pesquisa.

De Souza et al.^(26)^ iniciaram sua pesquisa a partir das atividades realizadas por enfermeiros em Ambulatório de Quimioterapia de um hospital especializado em Oncologia. Utilizaram como métodos: a entrevista semiestruturada, a análise de documentos e o questionário. Na sequência, foram mapeadas as atividades em intervenções, segundo a NIC.

As intervenções mapeadas neste estudo resultaram em 32 intervenções distribuídas e 6 domínios: Fisiológico complexo, Fisiológico básico, Comportamental, Sistema de saúde, Família e Segurança. A não utilização das atividades pessoais deveu-se ao fato de se considerar que as mesmas podem dificultar a comparação com outros estudos, já que podem variar entre instituições; entretanto, na oficina, foi sugerida a inserção da atividade pessoal, sem descrever o tipo.

Outros pesquisadores^(26)^ diferem em seus resultados. Em centro de quimioterapia, foram identificadas 35 intervenções e 48 atividades; dentre elas, estava a atividade pessoal, descrita como pausas para alimentação e atendimento às necessidades fisiológicas. As intervenções foram organizadas em cinco domínios e, ao contrário deste estudo, não foi identificado o domínio família. Já pesquisadores^(28)^ em centro de diagnóstico por imagem identificaram 32 intervenções e 92 atividades.

Durante a oficina, foi percebida a importância do equilíbrio da associação do conhecimento da NIC com a vivência prática. Esse fato também foi observado por outras autoras,^(28)^ que também identificaram a vantagem dessa técnica ao proporcionar uma análise crítica para o aperfeiçoamento do instrumento proposto.

As 25 intervenções e 1 atividade resultantes da validação da oficina compuseram um instrumento de medida de carga de trabalho, sendo as intervenções de Enfermagem listadas em ordem alfabética com seus respectivos códigos NIC. A jornada de trabalho foi dividida em colunas e intervalos de 10 minutos. Foram inseridos campos para colocação de até seis observadores. Essa mesma formatação já foi utilizada^(28)^ para propor um instrumento de medida da distribuição de tempo de trabalho dos profissionais de Enfermagem em centro de diagnóstico por imagem, porém não foi disponibilizado campo no instrumento para seis observadores simultâneos, reservando espaço para apenas dois deles.

## CONCLUSÃO

Ficou evidenciado que a utilização do referencial, a partir das intervenções essenciais para as áreas de especialidades de Enfermagem em Oncologia Pediátrica e Enfermagem Oncológica, otimizou o tempo da pesquisa. Entretanto, outras áreas de especialidades devem ser avaliadas como possibilidades de inserção na construção do instrumento, tais como Enfermagem Ambulatorial, Enfermagem de Cuidados Paliativos, Enfermagem em Saúde Ocupacional e Enfermagem na Emergência.

O instrumento desenvolvido foi validado em uma oficina, na qual demonstrou-se que ele era apropriado para mensurar a carga de trabalho da equipe de Enfermagem de Ambulatório de Oncologia e Hematologia. Entretanto, ele apresentou limitação, pois necessita ser aplicado na prática assistencial para avaliação de sua confiabilidade e, assim, definição do grau de precisão, ou seja, do quanto consegue refletir os dados identificados no instrumento proposto.

**Quadro 2:** Relação das 32 intervenções de Enfermagem selecionadas conforme a Classificação das Intervenções de Enfermagem (NIC) para o Ambulatório de Oncologia e Hematologia

Código	Intervenções mapeadas	Definições
8190	Acompanhamento por telefone	Oferecimento de resultados de exames e avaliação da reação do paciente, além de determinação do potencial de problemas, em consequência de tratamento, exames ou testes anteriores, usando o telefone
2210	Administração de analgésicos	Uso de agentes farmacológicos para reduzir ou eliminar a dor
4030	Administração de hemoderivados	Administração de sangue ou derivados do sangue e monitoramento da reação do paciente
2313	Administração de medicamentos intramuscular	Preparo e aplicação de medicamentos pela via intramuscular
2304	Administração de medicamentos oral	Preparo e administração de medicamentos pela boca
2314	Administração de medicamentos endovenosa	Preparo e aplicação de medicamentos pela via endovenosa
7040	Apoio ao cuidador	Oferecimento das informações necessárias, defesa e apoio para facilitar o cuidado primário ao paciente por pessoa que não seja um profissional de saúde
5420	Apoio espiritual	Assistência ao paciente para que tenha equilíbrio e conexão com poder superior
4430	Brinquedo terapêutico	Uso proposital e orientado de brinquedos, ou de outros materiais, para ajudar as crianças a comunicarem sua percepção e conhecimento do mundo e auxiliar a dominarem seu ambiente
1400	Controle da dor	Alívio da dor ou sua redução a um nível de conforto aceito pelo paciente
0590	Controle da eliminação urinária	Manutenção de um padrão excelente de eliminação urinária
1450	Controle da náusea	Prevenção e alívio da náusea
1100	Controle da nutrição	Auxílio ou oferta de ingestão nutricional equilibrada de alimentos e líquidos
2240	Controle da quimioterapia	Assistência ao paciente e aos familiares para que compreendam a ação e a minimização dos efeitos colaterais dos agentes antineoplásicos
6540	Controle de infecção	Minimizar a aquisição e a transmissão de agentes infecciosos
2380	Controle de medicamentos	Facilitar o uso seguro e eficaz de medicamentos prescritos e não prescritos
6482	Controle do ambiente: conforto	Manipulação do ambiente ao redor do paciente para promover o máximo de conforto
1570	Controle do vômito	Prevenção e alívio do vômito
4120	Controle hídrico	Promoção do equilíbrio hídrico e prevenção de complicações decorrentes de níveis anormais ou indesejados de líquidos
2080	Controle hidroeletrolítico	Regulação e prevenção de complicações decorrentes de níveis alterados de líquidos e/ou eletrolíticos
5230	Melhora do enfrentamento	Assistência ao paciente para se adaptar a estressores, mudanças ou ameaças percebidos, que interfiram na satisfação das exigências da vida e no desempenho de papéis
5566	Orientação aos pais: educando os filhos	Assistência aos pais para compreenderem e promoverem o crescimento e o desenvolvimento psicológico, físico e social do filho pequeno, do pré-escolar e do filho/filhos em idade escolar
4235	Punção de vaso cateterizado: amostra de sangue	Aspiração de amostra de sangue através de cateter vascular já inserido para exames laboratoriais
5820	Redução da ansiedade	Redução da apreensão, do receio, do pressentimento ou do desconforto relacionados a uma fonte não identificada de perigo antecipado
5880	Técnica para acalmar	Redução da ansiedade em paciente com sofrimento agudo
4200	Terapia endovenosa	Administração e monitoramento de líquidos e medicamentos endovenosos
3740	Tratamento da febre	Controle do paciente com hiperpirexia causada por fatores não ambientais

**Tabela 1:** Caracterização dos juízes participantes da oficina para validação das intervenções selecionadas segundo a Classificação das Intervenções de Enfermagem (NIC) em 4 de fevereiro de 2013. São Paulo, 2013

Características	n (%)
Sexo	
Feminino	5 (83,3)
Masculino	1 (16,7)
Idade	
≥30 anos	6 (100,0)
Categoria profissional	
Professor	3 (50,0)
Gerente de Enfermagem	1 (16,7)
Enfermeiro sênior	1 (16,7)
Técnico em Enfermagem	1 (16,7)
Maior titulação	
Doutorado	2 (33,3)
Mestrado	2 (33,3)
Especialização	1 (16,7)
Graduação em Enfermagem	1 (16,7)
Tempo médio de formado (ano)	
<1	1 (16,7)
≥ 5-10	1 (16,7)
≥10	4 (66,7)
Tempo médio de experiência em Oncologia (ano)	
0	2 (33,3)
7	1 (16,7)
10	1 (16,7)
12	1 (16,7)
15	1 (16,7)
Tempo médio de experiência NIC (ano)	
0	1 (16,7)
<1	2 (33,3)
6	1 (16,7)
7	2 (33,3)
